# N-acetylcysteine for patients with alcohol use disorder, post-traumatic stress disorder, and their co-occurrence: a systematic review of placebo-controlled randomized trials

**DOI:** 10.1186/s12888-026-08124-8

**Published:** 2026-05-11

**Authors:** Mohamed Awad E. Ahmed, Mufreh Amin, Yomna Emad Abdalla, Amr Abdelghani, Nourhan Eid, Aya Samy, Omar Kassar, Khalid Radwan Alsaadany, Mohamed Ezzat M. Mansour

**Affiliations:** 1 Faculty of Medicine, Suez University, Suez city, 43511 Egypt; 2 Faculty of Medicine, Misr University of Science and Technology, 6th of October City, Egypt; 3 Faculty of Medicine, Zagazig University, Zagazig city, Egypt; 4 Faculty of Medicine, Alexandria University, Alexandria city, Egypt; 5 Faculty of Medicine, Mansoura University, Mansoura city, Egypt

**Keywords:** Post-traumatic stress disorder, Alcohol use disorder, N-acetylcysteine

## Abstract

**Introduction:**

Alcohol use disorder (AUD) and post-traumatic stress disorder (PTSD) are thought to be significant global contributors to the burden of mental illness. According to recent WHO epidemiological estimates, 3.9% of people worldwide have experienced PTSD at some point in their lives; this number rises to roughly 5.6% among those who have experienced trauma. NAC has shown promise in reducing PTSD symptoms and cravings in veterans, according to recent trials. Our analysis aims to resolve the conflict between results and determine whether NAC is an effective add-on therapy for PTSD, AUD, and co-occurring PTSD/AUD.

**Methods:**

We conducted this study in accordance with the Preferred Reporting Items for Systematic Reviews and Meta-Analyses (PRISMA) statement, following our protocol (CRD420251170956). We searched PubMed, Scopus, Cochrane CENTRAL, Embase, EBSCO, and the Web of Science for relevant studies. Only randomized placebo-controlled trials were included. The primary outcomes were severity of PTSD and alcohol craving, while secondary outcomes were anxiety, depression, and alcohol use patterns and biomarkers.

**Results:**

Seven randomized, double-blind, placebo-controlled trials were included in this meta-analysis, enrolling a total of 566 participants. There were no significant differences between NAC and placebo in craving (SMD= -0.40, 95% CI [-1.05, 0.25], *P* = 0.22, I² = 56.1%). Regarding PTSD severity, the PCL scale pooled analysis demonstrated no significant difference between NAC and placebo (SMD = 0.04, 95% CI [-0.40, 0.48], *P* = 0.8) (I² = 65.9%, *P* = 0.05), while the CAPS scale pooled analysis demonstrated no significant difference between NAC and placebo (SMD = -0.13, 95% CI [-0.50, 0.24], *P* = 0.49) (I² = 54.8%, *P* = 0.109). Leave-one-out sensitivity analysis showed that exclusion of the Back et al. (2025) study resulted in a significant effect favoring NAC (SMD = -0.35, 95% CI [-0.70, -0.01], *P* = 0.04), with no observed heterogeneity (I² = 0%).

**Conclusion:**

Our meta-analysis indicates that N-acetylcysteine, when compared to placebo, does not demonstrate consistent efficacy in reducing the primary symptoms of AUD or PTSD in broad patient populations. However, the treatment is relatively safe. A noteworthy signal of potential efficacy for PTSD symptoms emerged in a sensitivity analysis, suggesting that the therapeutic promise of NAC should not be entirely dismissed.

**Clinical trial number:**

Not applicable.

**Supplementary Information:**

The online version contains supplementary material available at 10.1186/s12888-026-08124-8.

## Introduction

Post-traumatic stress disorder (PTSD) and alcohol use disorder (AUD) are considered major contributors to the burden of psychiatric disease worldwide [1]. Recent epidemiological estimates of the WHO reported that 3.9% of the population globally developed post-traumatic stress disorder (PTSD) at some stage in their lives, increases to approximately 5.6% among trauma-exposed individuals [2]. Concurrently, it was reported that around 7% of adults aged 15 years and older meet criteria for an alcohol use disorder, accounting for over 400 million people worldwide [3]. According to epidemiological surveys, comorbidity between PTSD and AUD is remarkably high, about 30 to 50% of individuals with PTSD also match criteria for a substance use disorder, most commonly AUD [4]. This association results in worse treatment outcomes, severe symptoms, and higher relapse rates, shedding light on the urgent need for novel medications that target common neurobiological substrates [5].

Pathophysiologically, PTSD and AUD are characterized by dysregulations of fear circuitry, stress reactivity, and glutamatergic signaling accompanied by elevated oxidative stress [6]. Individuals with PTSD exhibit sustained hyperactivation of the amygdala and disturbances of the prefrontal-limbic inhibitory network, associated with elevated neuroinflammatory signaling [7]. Beyond traditional models, recent evidence highlights that severe stress exposure, such as war-related trauma, can trigger profound depressive reactions and post-traumatic symptoms through a cascade of inflammatory activation, mitochondrial dysfunction, and redox imbalance [8]. Similarly, AUD induces pathological alterations in cortico-striatal circuits, imbalance of glutamate–γ-aminobutyric acid (GABA) receptors, and mitochondrial oxidative injury that raises vulnerability for craving and relapse [9]. Preclinical and clinical studies proved that glutamate homeostasis and antioxidant defences are the main overlapping mechanisms of both conditions that should be targeted by pharmacological therapy [9].

NAC acts as a cysteine prodrug, helping in the enhancement of glutamate transporter expression and function which restores central nervous system homeostasis. It works via stimulating the cystine–glutamate antiporter, increasing extracellular glutamate, and activating inhibitory mGlu2/3 receptors [10].This reduces excessive excitatory tone within cortico–striatal–limbic circuits implicated in craving, stress reactivity, and hyperarousal [11]. Furthermore, NAC has a major role in reducing oxidative damage, neuroinflammation pathways, and mitochondrial dysfunction through replenishing glutathione, an amino acid considered a primary endogenous antioxidant responsible for cellular oxidative balance [12].

Recent trials have reported a promising effect of NAC in improving PTSD symptoms and craving in veterans [13, 14]. Moreover, a randomized, double-blind, placebo-controlled clinical trial was conducted on 60 patients with SUD under MMT who received NAC as an additional therapy for 12 weeks, Padoei et al., 2023 reported significant improvement in depression score, anxiety levels, and elevation of total antioxidant capacity levels [15]. Although limited evidence directly targeted the effect of NAC on co-occurring PTSD and AUD, (Back et al., 2025) found that NAC showed good tolerability and reductions in craving symptoms, but results did not reach statistically significant group differences [14, 16].

Meta-analyses have also shown inconsistent results regarding the effect on craving and abstinence; Duailibi et al. (2017) and Chang et al. (2021) [17, 18] reported superior effects of NAC on craving, whereas Winterlind et al. (2024) [19] found no significant advantage over placebo (SMD = 0.189, 95% CI = − 0.015–0.393). These meta-analyses were limited by small sample sizes and high heterogeneity and high publication bias.

To date, no systematic review has collected RCT evidence assessing NAC for PTSD, AUD, and their co-occurrence. The present research considers the first systematic review and meta-analysis to critically appraise and accurately investigate the efficacy and safety of using NAC in these conditions. Our analysis aims to resolve the conflict between results and determine whether NAC is an effective add-on therapy. Identifying this gap in knowledge will provide future research with data regarding this promising pharmacologic therapy.

## Methods

### Protocol and registration

We followed the PRISMA statement guidelines in this systematic review and meta-analysis [20]. We conducted this study based on the Cochrane Handbook of Systematic Reviews of Interventions [21]. This study was prospectively registered on PROSPERO (CRD420251170956).

### Criteria for considering studies in this review

Studies satisfying the following inclusion criteria were included in the systematic review:

- Population: patients with a primary diagnosis of alcohol use disorder (AUD), post-traumatic stress disorder (PTSD), or co-occurring AUD and PTSD, diagnosed according to DSM-5 or DSM-4 criteria.
- Intervention: studies where the experimental group received NAC (all doses were eligible).
- Comparator: studies where the control group received a placebo.
- Outcomes: The primary outcomes were Severity of PTSD (using The Clinician-Administered PTSD Scale (CAPS) and The PTSD Checklist (PCL)) and Alcohol Craving. The secondary outcome measurements were Anxiety, Depression, and Alcohol Use Patterns and Biomarkers.
- Study design: studies that were described as RCTs.

We excluded articles that were case reports/case series, thesis, conference abstracts, animal studies, secondary studies, studies on schizophrenia and bipolar disorders.

### Literature search and keywords

We searched PubMed, Scopus, Cochrane CENTRAL, EBSCO, Embase, and the Web of Science for relevant studies till 14/10/2025. The search strategy was:

((N-acetyl cysteine) OR (NAC) OR (Acetylcysteine)) AND ((Posttraumatic stress disorder) OR (PTSD) OR (Alcohol use disorder) OR (ethanol consumption) OR (Alcohol consumption) OR (alcohol preference) OR (Ethanol preference) OR (Alcohol abuse) OR (Ethanol abuse) OR (Alcohol dependence) OR (Ethanol dependence) OR (Alcoholism)).

### Screening and study selection process

A literature search and screening were done independently by three authors (MA, AA, and NE) and conflicts were resolved by (MAA). Eligibility screening was done using Rayyan tool [22]. Studies were screened in two levels. The first level was screening the title and abstract to ensure matching the inclusion criteria. In the second level, we checked the full-text articles for eligibility to our meta-analysis criteria.

### Data extraction

Four authors (YE, AA, NE, and AS) extracted the data independently using an online data extraction form. The extracted data were mainly divided into four domains: [1] study characteristics [2], characteristics of the included study population [3], risk of bias domains, and [4] study outcomes. Data was exported as a Microsoft Excel sheet, and authors (MAA and MEM) resolved disagreement.

### Assessment of the risk of bias in included studies

We assessed the quality of each included study using the Cochrane risk of bias (ROB-2) tool 2. The Cochrane ROB-2 tool evaluates the risk of bias in five domains: [1] bias arising from the randomization process [2], bias due to deviations from intended intervention [3], bias due to missing outcome data [4], bias in measurement of the outcome, and [5] bias in selection of the reported result. Each study was tagged as ‘low risk,’ ‘high risk’ or ‘some concerns’ after careful revision of the data presented in the published articles.

### Measures of treatment effect

The primary outcomes were Severity of PTSD and Alcohol Craving Measures.

#### Severity of PTSD

The Clinician-Administered PTSD Scale for DSM-5 (CAPS-5) is a standardized 30-item interview that serves as the primary diagnostic tool for PTSD by allowing both diagnosis and measurement of symptom severity, using ratings from 0 to 4 for frequency and intensity [23]. 

The PTSD Checklist for DSM-5 (PCL-5) includes 20 items that measure DSM-5 symptoms, providing a total severity score (0–80) and cluster scores, allowing for provisional diagnosis with item ratings of 2 or higher, based on DSM-5 criteria [23].

The PTSD Checklist–Military Version (PCL-M) consists of 17 items which focus on military-related PTSD symptoms that occur during stressful military events. The assessment uses a 1 to 5 rating system where participants evaluate each item from (not at all) to (extremely). The PCL-M provides scores which range from 17 to 85 according to the total assessment outcomes[24].

#### Alcohol craving measures

The Penn Alcohol Craving Scale (PACS) is a five-item, self-report measure administered weekly that assesses frequency, intensity, and duration of craving thoughts, resistance to drinking, and overall craving intensity on a 0–6 scale per item[25].

The Alcohol Urge Questionnaire (AUQ) is an eight-item instrument created to capture immediate urge-to-drink experiences and drinking-related phenomena in the present moment [26].

The Visual Analogue Scale (VAS) is widely used in addiction research as a rapid measure of subjective craving intensity and is sensitive to short-term fluctuations. It features a 100-mm line for participants to rate their craving strength from “not at all” to “extremely. The final score is calculated as the distance in millimeters from the left anchor (0–100) [25].

The secondary outcome measurements were Anxiety, Depression, and Alcohol Use Patterns and Biomarkers.

#### Anxiety and depression

The Hospital Anxiety and Depression Scale (HADS) is a 14-item self-report measure developed for general hospital outpatients, comprising two independent 7-item subscales for anxiety and depression, each scored 0–21 using 4-point response options referring to the past week; it deliberately minimizes somatic content and uses conventional ranges of 0–7 (normal), 8–10 (borderline), and ≥ 11 (probable case) [27].

The Beck Depression Inventory (BDI-II) contains 21 items which enable patients to assess their depressive symptoms based on DSM-IV major depressive episode criteria from the past two weeks. It assesses depression through two separate factors which evaluate cognitive–affective symptoms and somatic–vegetative symptoms [28].

The Depression Anxiety Stress Scales (DASS) is a 42 self-report instrument with three 14-item scales (Depression, Anxiety, Stress) that ask participants to evaluate their symptoms from 0 to 3 based on their experiences during the previous week. It maintains a stable three-factor structure which researchers have proven through various studies conducted with both clinical and non-clinical participants [29].

#### Alcohol use patterns and biomarkers

The Timeline Follow back (TLFB) is a calendar-based, retrospective method in which individuals reconstruct their day-by-day alcohol use over a defined interval, using anchors and memory aids to enhance recall and generate detailed estimates of drinking patterns [30].

Drinks per drinking day: Using TLFB, trials on alcohol use disorder and co-occurring PTSD/AUD calculate “average number of standard drinks per drinking day” as a primary outcome, reflecting the typical intensity of drinking on days when alcohol is consumed. TLFB allows this mean drinks-per-drinking-day index to be tracked over time, making it sensitive to treatment-related reductions in drinking intensity in both general AUD samples and comorbid populations [31].

Heavy drinking days: TLFB-based studies routinely define heavy drinking days as those meeting sex-specific thresholds (e.g., ≥ 4 standard drinks for women, ≥ 5 for men) and count their frequency over weeks or months to capture high-risk drinking. Methodological work shows that this day-by-day TLFB approach detects sporadic and binge-level heavy drinking much more accurately than traditional quantity–frequency summaries, which often underestimate or obscure such episodes [14].

Liver enzyme biomarkers: GGT, AST, and ALT are indirect liver enzymes that reflect chronic alcohol-related liver injury but are not specific for alcohol use disorder [32].

- GGT: Membrane-bound enzyme involved in glutathione and bile metabolism; rises after several weeks of heavy drinking ( ≈ ≥ 60–74 g/week), normalizes within 2–6 weeks of abstinence, and is sensitive but poorly specific because many non-alcoholic conditions and drugs also elevate it [33].
- AST/ALT: Hepatocellular enzymes; in alcohol-associated liver disease AST is typically higher than ALT (AST/ALT ≥ 1–2), supporting an alcohol-related pattern, but both are non-specific and can be raised in many other hepatic and systemic diseases [34].

### Data synthesis

All statistical analyses and plotting were conducted using RStudio software, R version 4.2.2 [34, 35]. The “meta” package was used to calculate the effect size and generate forest plots. The mean difference (MD) and standardized mean difference (SMD) were adopted as the effect estimate with a 95% confidence interval (CI) and we prioritized the use of mean and standard deviation of change from baseline. Mean drinks per drinking day were pooled as MD in a meta-analysis model. Changes in CAPS, PCL, Alcohol craving, Heavy drinking days, liver enzymes, depression and anxiety were pooled as (SMD). All safety outcomes, dichotomous data from prospectively designed studies, were reported as risk ratio (RR) between the NAC and control group. We used the random-effects model for all outcomes, which accounts for both within-study and between-study variance (tau2). In the case of studies reporting data at multiple time points, we considered the last endpoint for primary analysis. When the standard deviation (SD) of change of included outcomes was not provided, we calculated it from the standard error or 95% confidence interval (CI) according to Altman[35].

### Assessment of heterogeneity

Statistical heterogeneity among studies was evaluated by the Chi-square test (Cochrane Q test) and the I2 statistic. I2 was interpreted as the proportion of total variation across studies attributable to heterogeneity. A Chi-square P value less than 0.1 and I2 > 50% was considered a significant heterogeneity. The restricted maximum likelihood (REML) estimator was used to calculate tau2 as a measure of the magnitude of between-study heterogeneity. Sensitivity analysis were performed to resolve heterogeneity.

#### Leave-one-out sensitivity analysis

Primary Leave-one-out sensitivity analyses were conducted for two outcome measurements, CAPS and PCL.

## Results

### Results of literature search

**Fig. 1:**
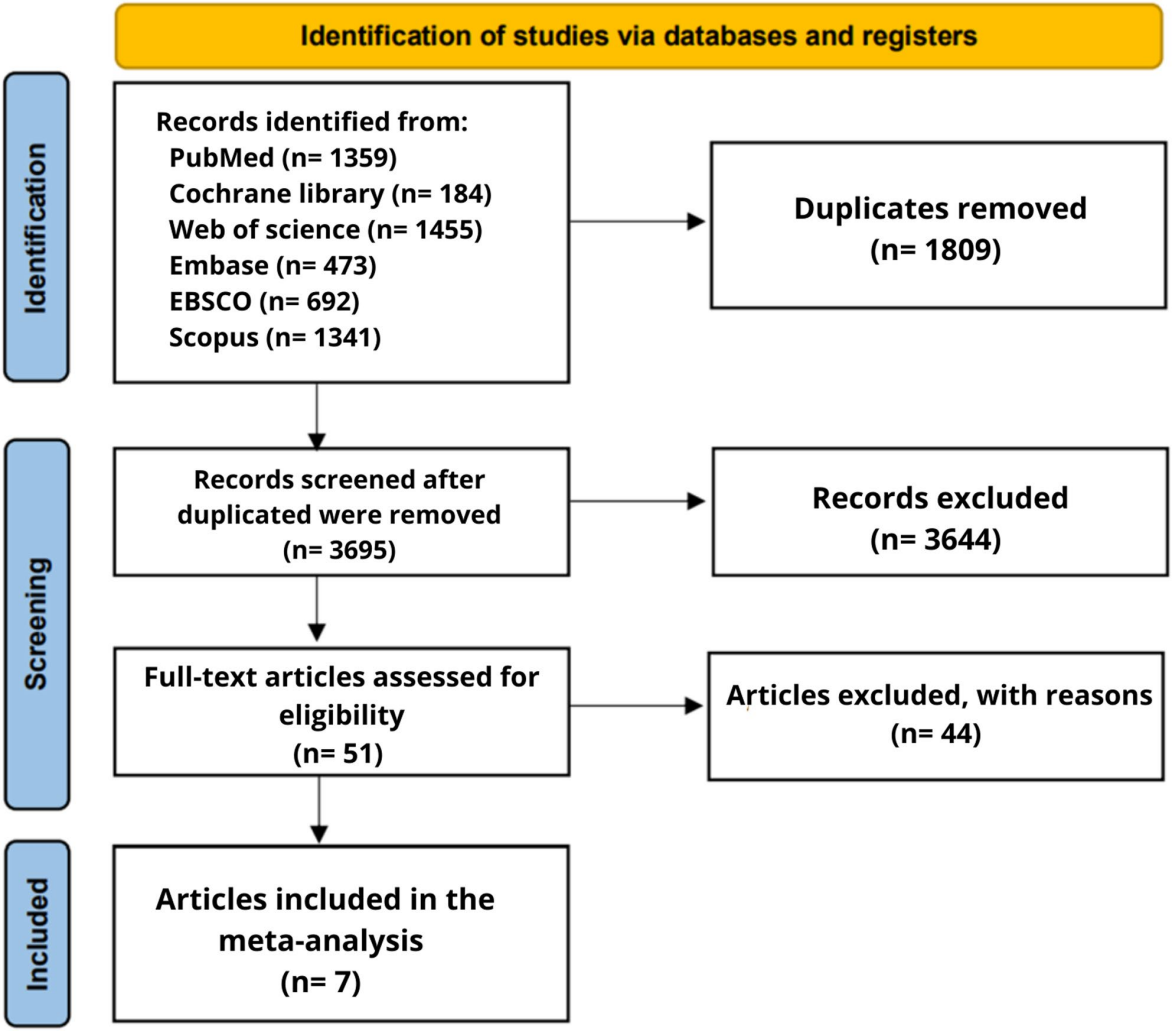
Flow diagram of the study selection process

The process of the literature search included 5504 unique studies. three authors (MA, AA, and NE) screened records. Any conflicting error was resolved by the author (MAA). Of them,1809 were identified as duplicates by Rayyan. Fifty-one full-text articles were reviewed and screened for the eligibility criteria. Of them, seven studies were included in this study. The PRISMA flow diagram of the study selection process is shown in Fig. 1.

### Characteristics of the included studies

Seven randomized, double-blind, placebo-controlled trials evaluating N-acetylcysteine (NAC) were included in this meta-analysis, enrolling a total of 566 participants. Studies ranged in sample size from 23 to 182, with most trials recruiting adults aged 18–65 years; one study focused on adolescents and young adults aged 15–25. Across studies, participants were diagnosed with alcohol use disorder (AUD), post-traumatic stress disorder (PTSD), or both, using DSM-IV or DSM-5 criteria. Populations included treatment-seeking individuals, as well as those with moderate-to-severe, treatment-resistant, or comorbid psychiatric disorders.

The intervention comprised oral NAC at daily doses between 1200 mg and 2700 mg, compared with matching placebo for durations ranging from 4 to 12 weeks. Included studies were conducted in outpatient mental health clinics, addiction units, and academic hospitals in the United States, Australia, and Brazil. All trials employed validated outcome measures and were assessed using the ROB-2 tool; Five trials were rated as low risk of bias, one as some concerns, and one as high risk of bias. Comprehensive details of study populations, trial design, interventions, and outcome measures are summarized in Table 1.

**Table 1:** Characteristics of the included studies

Study	Registration number	Population	Sample Size	Disease	Severity of the disease	Intervention	Comparator	Dose or Target dose	Duration	Location	Results
Back 2016 [13]	NCT02499029	35 Veterans, 18–65 years, with PTSD & SUD	35	PTSD, AUD	PTSD or subthreshold PTSD	NAC	Placebo	2400 mg/day	8 weeks	USA	NAC led to greater relief of PTSD symptoms and cravings; substance use was low in both groups.
Kanaan 2023 [36]	ACTRN12618001784202	105 adults,18–65 years, unresponsive to first-line PTSD therapies	105	PTSD	Refractory to standard treatment	NAC	Placebo	2.7 g/day	12 weeks	Australia	No significant differences at 12 weeks; longer-term craving control favored NAC at 64 weeks.
Back 2025 [14]	NCT02966873	182 adults, 18–65 years, treatment-seeking for PTSD & alcohol use	182	PTSD, AUD	Moderate to severe comorbidity	NAC	Placebo	2400 mg/day	12 weeks	USA	NAC was safe, but did not provide added improvement in PTSD or alcohol use compared to placebo.
Logge 2024 [37]	NCT03879759	23 adults with clinically significant alcohol misuse	23	AUD	Moderate to severe	NAC	Placebo	2400 mg/day	4 weeks	Australia	NAC affected some brain connectivity patterns but showed no benefit for alcohol craving or cue response.
Morley 2023 [31]	NCT03879759	Adults, 18–65 years, DSM-5 alcohol use disorder	42	AUD	Variable severity	NAC	Placebo	2400 mg/day	28 days	Australia	No clinical advantage seen with NAC in alcohol use or retention compared to placebo.
Schuch 2024 [38]	NCT03018236	53 adults, 18–65 years with AUD diagnosed by the DSM-5.	53	AUD	Mostly moderate	NAC (as an adjuvant therapy)	Placebo	1,200 mg/day	8 weeks	Brazil	NAC altered certain biomarkers but had no effect on clinical outcomes such as relapse or adherence.
Squeglia 2025 [39]	NCT03707951	Adolescents/young adults, 15–25 years in outpatient care	126	AUD	Moderate to severe	NAC	Placebo	2400 mg/day	8 weeks	USA	NAC did not reduce drinking or improve secondary outcomes over placebo.

### Risk of bias of included studies

**Fig. 2:**
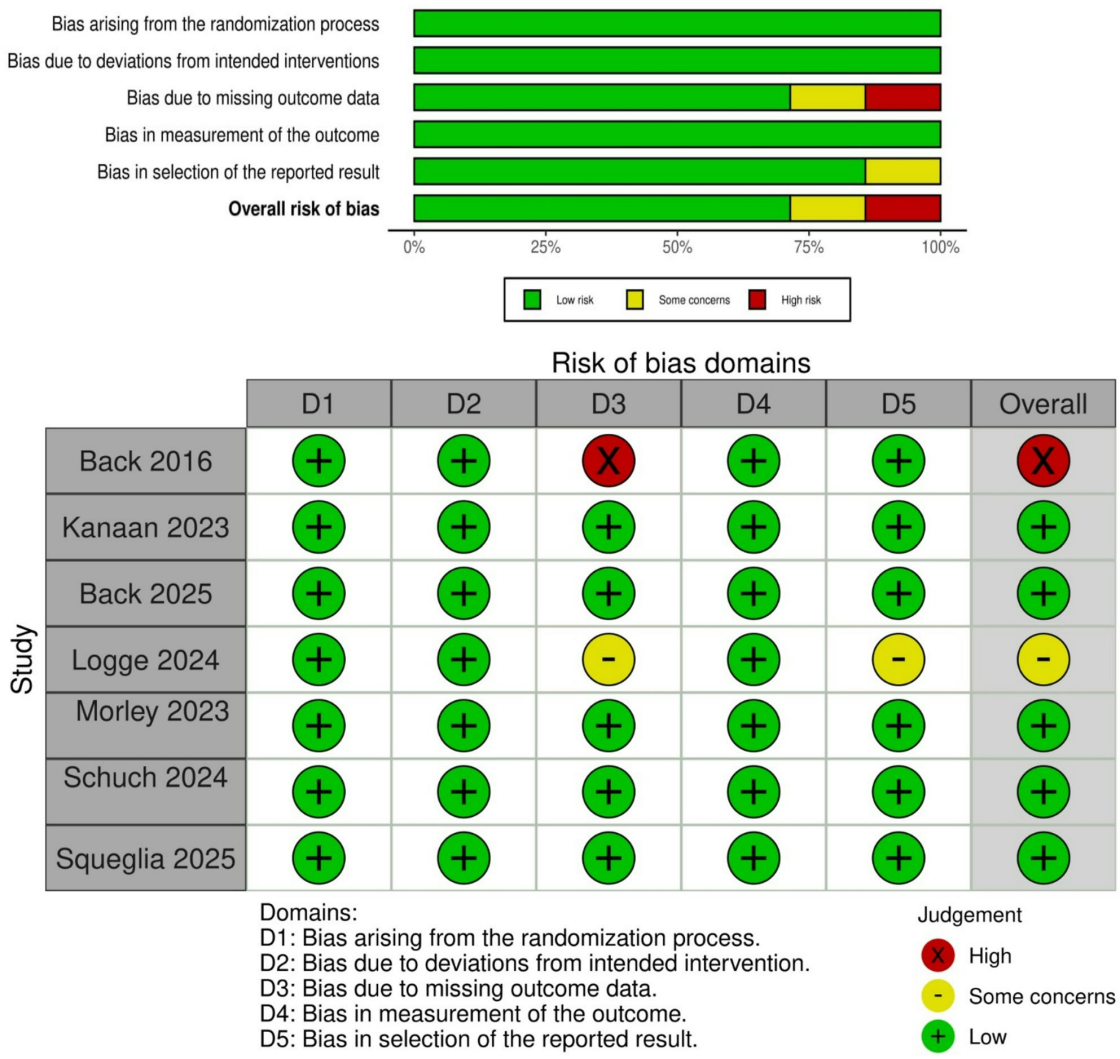
Quality assessment using Cochrane risk of bias tool (**A**) Traffic light plot and (**B**) Summary of risk of bias assessment

Two authors (MA and KRA) independently assessed the quality of each included study according to Cochrane Handbook of Systematic Reviews of Interventions. Among the included studies, one study was assessed as having a high risk of bias, one study was rated as some concerns, and five were determined to have a low risk of bias. A summary of quality assessment domains is shown in Fig. 2**.**

### Meta-analysis of NAC efficacy in AUD

#### Craving outcomes

**Fig. 3:**
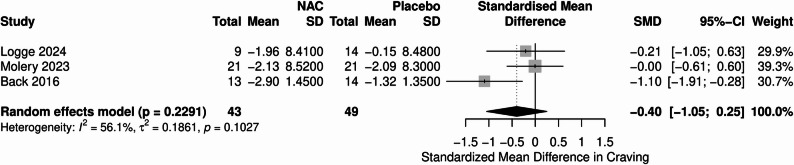
Forest plot illustrating alcohol craving

Three studies with 92 patients reported the effects of NAC on alcohol craving. The pooled analyses demonstrated no significant differences between NAC and placebo in craving (SMD= -0.40, 95%CI [-1.05, 0.25], *P* = 0.22, I²= 56.1%) **(**Fig. 3**).**

#### Drinking behaviors

For the heavy drinking days, the pooled analysis of 168 patients showed no significant difference between groups (SMD = 0.21, 95%CI [-0.6, 1.02], *P* = 0.6) (Fig. Supplementary 1), with Significant heterogeneity (I² = 80.7%, *P* = 0.022). Regarding mean difference in average drinks per drinking day, there was no significant difference between NAC and placebo (MD = 0.12, 95%CI [-0.83, 1.06], *P* = 0.8, I²= 0%) (Fig. Supplementary 2).

#### Liver enzymes

Two studies including 95 patients reported changes in liver parameters. The pooled analysis showed no significant differences between NAC and placebo in ALT (SMD= -0.47, 95% CI [-2.18, 1.23], *P* = 0.58, I²= = 93.5%), AST (SMD= -0.26, 95%CI [-1.33, 0.81], *P* = 0.63, I2 = 85%), or GGT (SMD = -0.20, 95% CI [-0.61, 0.20], *P* = 0.33, I² = 0%) (Fig. Supplementary 3).

### Meta-analysis of NAC efficacy in PTSD

**Fig. 4:**
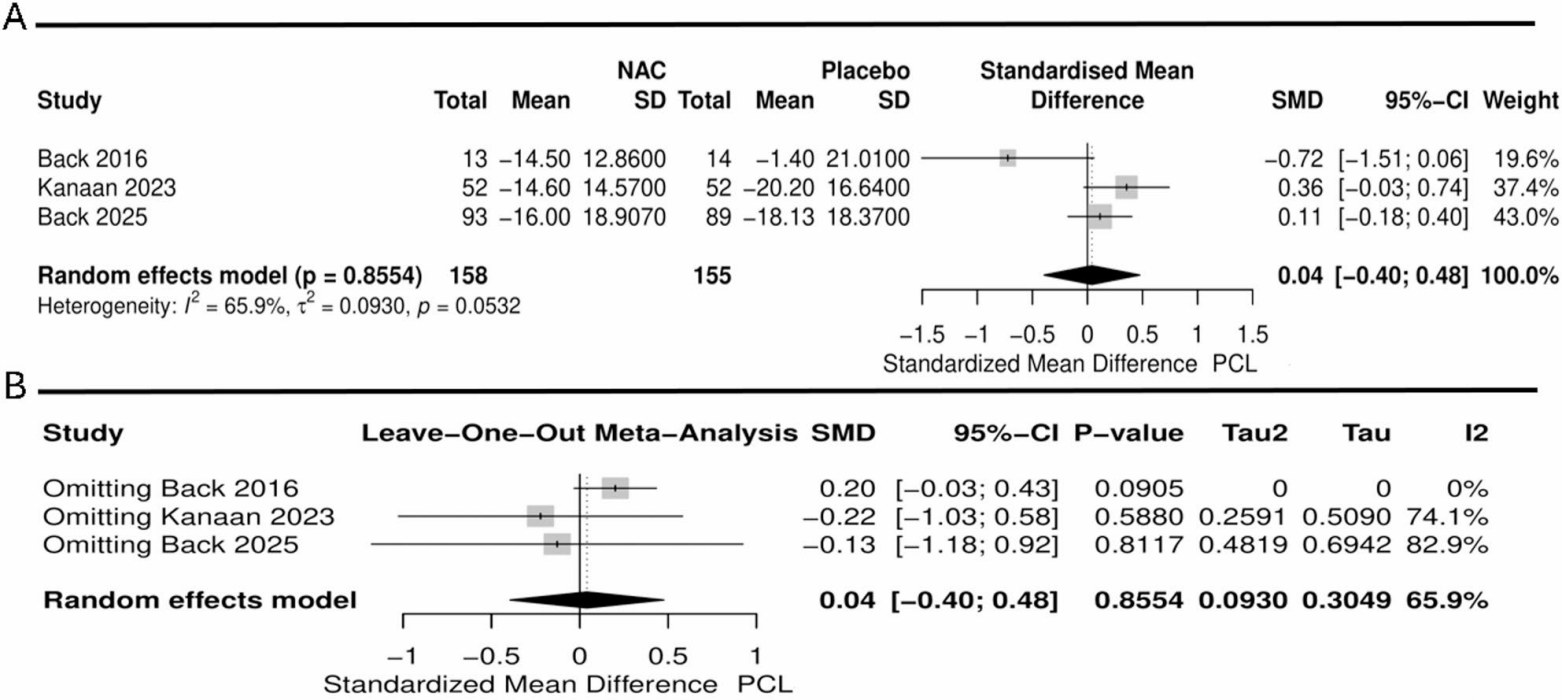
Forest plot illustrating (**A**) PCL scale and (**B**) Leave-one-out sensitivity analysis of PCL scale

**Fig. 5:**
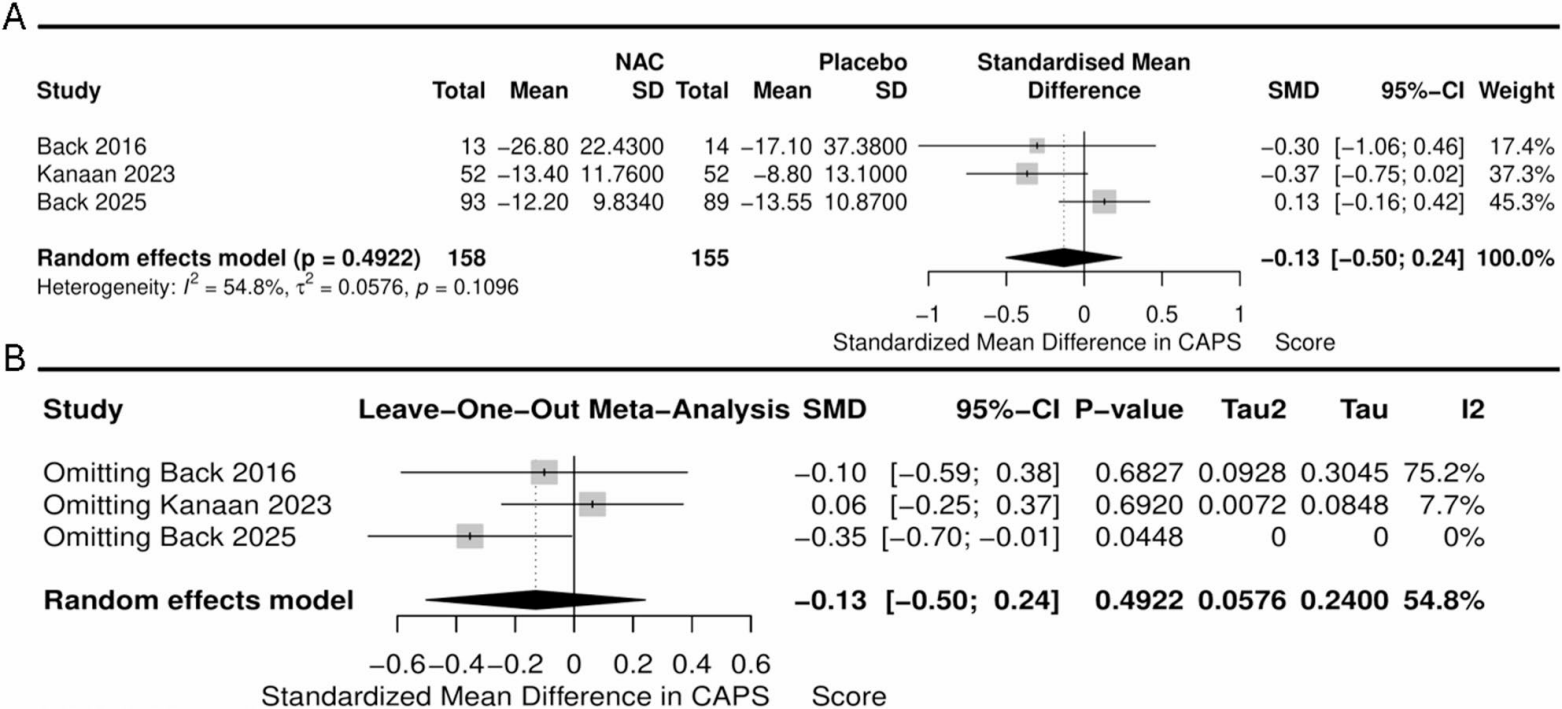
Forest plot illustrating (**A**) CAPS scale and (**B**) Leave-one-out sensitivity analysis of CAPS scale

Three studies including 313 patients reported changes in PTSD symptom severity using the PCL scale. The pooled analysis demonstrated no significant difference between NAC and placebo (SMD = 0.04, 95%CI [-0.40, 0.48], *P* = 0.8), with significant heterogeneity (I²= 65.9%, *P* = 0.05) (Fig. 4A). Leave-one-out sensitivity analysis revealed that the heterogeneity was resolved by excluding the Beck et al. (2016) study. However, the overall effect remained non-significant (SMD = 0.2, 95%CI [-0.03, 0.43], *P* = 0.09, I²= = 0)(Fig. 4B). Regarding CAPS scale, the pooled analysis demonstrated no significant difference between NAC and placebo (SMD= -0.13, 95%CI [-0.50, 0.24], *P* = 0.49), with no significant heterogeneity (I²= 54.8%, *P* = 0.109) (Fig. 5A). Leave-one-out sensitivity analysis for the CAPS scale showed that exclusion of the Back et al. (2025) study resulted in a significant effect favoring NAC (SMD= -0.35, 95% CI [-0.70, -0.01], *P* = 0.04), with no observed heterogeneity (I² = 0%) (Fig. 5B).

### Meta-analysis of depressive and anxiety symptoms

Three studies comprising 173 patients reported changes in depressive symptoms. The pooled analysis demonstrated no significant difference between NAC and placebo (SMD= -0.12, 95% CI [-0.48, 0.25], *P* = 0.52, I²= 24%) (Fig. Supplementary 4). Two studies reported anxiety symptoms, and the pooled effect also showed no significant difference between NAC and placebo (SMD= -0.13, 95%CI [-0.46, 0.19], *P* = 0.42, I²= 0%) (Fig. Supplementary 5).

### Meta-analyses of safety outcomes

Pooled analysis showed no significant difference between NAC and Placebo in discontinuation rates (RR = 1.22, 95%CI [0.69, 2.16], *P* = 0.499, I²= 0%, Fig. Supplementary 6 A), infections (RR = 0.96, 95%CI [0.67, 1.38], *P* = 0.82, I²= 0%, Fig. Supplementary 6 C), muscle or joint pain (RR = 0.78, 95%CI [0.37, 1.62], *P* = 0.5, I²= 0%, Fig. Supplementary 6D), or psychiatric symptoms (RR = 1.02, 95%CI [0.5, 2.08], *P* = 0.95, I²= 0%, Fig. Supplementary 6E). NAC showed a significantly higher risk for gastrointestinal symptoms than placebo (RR = 1.4, 95%CI [1.07, 1.84], *P* = 0.01, I²= 0%, Fig. Supplementary 6B).

## Discussion

### Summary of the key findings

Our systematic review and meta-analysis evaluated the therapeutic efficacy of N-acetylcysteine (NAC) across several psychiatric disorders. For Alcohol Use Disorder (AUD), the intervention failed to demonstrate a statistically significant effect on mitigating alcohol craving, reducing heavy drinking days, or normalizing hepatic enzyme profiles. Similarly, the primary pooled analysis for post-traumatic stress isorder (PTSD) did not reveal a significant treatment effect on symptom severity as measured by PCL and CAPS scales. Notably, CAPS leave-one-out sensitivity analysis excluding Back et al. 2025 suggested a modest benefit, indicating a need for further investigation. NAC also did not exhibit a significant therapeutic advantage over placebo for co-morbid depressive or anxiety symptoms. Regarding tolerability, NAC was generally well-tolerated, with discontinuation rates comparable to placebo, but with a higher incidence of gastrointestinal adverse events.

### Explanation of the study results

#### N-acetylcysteine in alcohol use disorder (AUD)

Our finding that NAC did not significantly impact alcohol craving or consumption patterns in the primary analysis contributes to a complex and often contradictory body of literature. The glutamatergic system, particularly the dysregulation of glutamate homeostasis in the nucleus accumbens, is a key neurobiological substrate implicated in the pathophysiology of addiction and relapse[16]. NAC is theorized to exert its therapeutic effects by restoring glutamate balance through the upregulation of the cystine-glutamate antiporter (system xc−) and glial glutamate transporter 1 (GLT-1), thereby reducing synaptic glutamate levels and mitigating drug-seeking behavior[40].

However, the clinical translation of this compelling preclinical evidence has been inconsistent. Some earlier meta-analyses reported a significant effect of NAC on reducing craving across various substance use disorders (SUDs)[17]. For instance, a review by Duailibi et al. found a large effect size in favor of NAC for craving reduction[17]. Conversely, more recent and comprehensive analyses have presented a more cautious view, suggesting that while NAC may reduce craving, the evidence is weak and hampered by significant heterogeneity[41]. Our results align more closely with this latter perspective and with findings from large-scale trials like the one reported by Back et al. 2025, which found no significant difference between NAC and placebo in reducing alcohol use or craving in patients with co-occurring AUD and PTSD [14]. The substantial heterogeneity in our analysis of heavy drinking days underscores the variability across studies, which may stem from differences in patient populations, NAC dosage, treatment duration, and the specific outcome measures used[19].

Regarding the non-significant effect on liver enzymes, this result is noteworthy given NAC’s established role as a hepatoprotective agent in acetaminophen overdose[16]. Its mechanism in that context involves replenishing glutathione (GSH) stores, a primary antioxidant. While chronic alcohol consumption also induces oxidative stress and depletes GSH in the liver, the pathophysiology of alcohol-associated liver disease is multifactorial, involving inflammation and metabolic dysregulation [42].

Our findings suggest that, within the timeframe and dosages used in the included trials, NAC supplementation did not translate into a measurable improvement in the liver function tests of patients with AUD. This may indicate that the participants did not have significant liver injury at baseline or that a more prolonged or higher-dose intervention is necessary to observe such effects. This aligns with the notion that while NAC is being explored for AUD, its specific role in treating associated liver injury remains an area for further research[43].

#### N-acetylcysteine in post-traumatic stress disorder (PTSD)

The results for PTSD present a particularly intriguing dichotomy. The primary analysis, showing no overall benefit of NAC, is consistent with some recent large-scale investigations. For example, a multi-center trial by Kanaan et al. (2023) for treatment-resistant PTSD found no significant difference between NAC (2.7 g/day) and placebo on the primary outcome (CAPS-5 score) at 12 weeks [36]. Similarly, the trial by Back et al. (2025) in comorbid AUD/PTSD also reported null findings for both conditions [14]. These results challenge the hypothesis that modulating glutamate and oxidative stress pathways with NAC is a broadly effective strategy for PTSD.

However, the significant result favoring NAC in our leave-one-out sensitivity analysis of CAPS scale data cannot be dismissed. This finding suggests that a single, potentially atypical study may disproportionately influence the overall null effect. The emergence of a significant impact (SMD = -0.35, *p* = 0.04) with the complete resolution of heterogeneity (I² = 0%) upon excluding the Back et al. (2025) study is a strong signal. This may indicate that NAC could be effective for a specific phenotype of PTSD not represented in the excluded study, or that methodological differences in that trial obscured a true treatment effect. For instance, the Back et al. (2025) study involved a comorbid AUD population receiving concurrent cognitive-behavioral therapy (CBT) for AUD, which could introduce confounding effects or represent a more treatment-resistant cohort[14]. In contrast, an earlier pilot study by Back et al. (2016) in veterans with PTSD and SUD found significant improvements in PTSD symptoms and craving with NAC[13].

Our sensitivity analysis result breathes new life into this earlier finding, suggesting that the therapeutic potential of NAC for PTSD remains a valid and important area of inquiry, particularly for non-comorbid populations or as an adjunct to different psychotherapies. The neurobiological rationale remains strong, as PTSD is associated with glutamatergic dysfunction and heightened oxidative stress, both of which are direct targets of NAC[44]. Furthermore, NAC should be viewed within an emerging translational framework that links systemic inflammatory states to neuropsychiatric manifestations. Recent research into metabolic modulators, such as NAC and acetyl-L-carnitine, suggests that targeting systemic oxidative stress may mitigate the neurobiological consequences of chronic inflammation, further supporting the investigation of these agents in stress-related disorders [45]. This is consistent with the broader evidence supporting anti-inflammatory agents as adjunctive therapies in neuropsychiatric conditions [46], reinforcing the rationale for targeting neuroinflammation in AUD and PTSD.

#### Effects on depressive and anxiety symptoms and safety profile

The lack of a significant effect of NAC on depressive and anxiety symptoms is somewhat surprising, given the high comorbidity of these symptoms with AUD and PTSD and the proposed mechanisms of NAC. Oxidative stress and inflammation are implicated in the pathophysiology of depression, and some meta-analyses have found that adjunctive NAC can ameliorate depressive symptoms, particularly in bipolar disorder[47]. However, other comprehensive reviews have concluded that NAC has no significant effect on depressive symptoms in major depressive disorder or bipolar disorder[48]. Our findings contribute to this latter body of evidence, suggesting that in the context of primary AUD or PTSD, the potential antidepressant or anxiolytic effects of NAC are not robust.

Finally, our safety analysis provides crucial clinical information. The finding that NAC significantly increases the risk of gastrointestinal symptoms is highly consistent across the literature and is considered the most common adverse effect of oral NAC administration[40]. This is an important factor for clinical practice, as it may affect treatment adherence. Reassuringly, we found no increased risk for other serious adverse events or higher discontinuation rates, confirming the generally favorable safety profile of NAC. This supports its potential use as an over-the-counter or adjunctive therapy, provided patients are counseled about the potential for gastrointestinal discomfort.

### Novelty of our study

Our meta-analysis provides a contemporary and focused synthesis of evidence that distinguishes itself from prior work in several ways. First, by concurrently evaluating the efficacy of NAC for both AUD and PTSD, our study addresses the significant clinical reality of their comorbidity. While previous meta-analyses have typically focused on a broader range of SUDs [18] or on PTSD as a singular condition, our dual focus allows for a more nuanced comparison of NAC’s effects across these interconnected disorders.

Second, our rigorous application of leave-one-out sensitivity analyses, particularly in the context of high heterogeneity, provides novel insights. The identification of a potentially significant effect for NAC on PTSD symptoms (via the CAPS scale) after the exclusion of a single influential trial is a key novel finding. This moves beyond a simple declaration of a null result and instead generates a specific, testable hypothesis about the conditions under which NAC might be effective, thereby guiding future research with greater precision.

Third, the inclusion of a comprehensive range of secondary outcomes, including liver function tests, depressive and anxiety symptoms, and a detailed safety profile, offers a more holistic view of NAC’s clinical utility than studies focused solely on primary substance use or trauma symptoms. By confirming the increased risk of gastrointestinal side effects while simultaneously showing no significant impact on liver enzymes or discontinuation rates in this specific population, our work provides a balanced and clinically relevant summary that can directly inform risk-benefit discussions between clinicians and patients.

### Strength and limitations

The strengths of this meta-analysis include a systematic and comprehensive literature search, adherence to established reporting guidelines, and the inclusion of multiple clinically relevant outcomes for both efficacy and safety. By employing rigorous statistical methods, including random-effects models to account for anticipated heterogeneity and sensitivity analyses to explore its sources, we have provided a robust evaluation of the available data. The separate pooling of data for different symptom scales and outcomes allowed for a more granular and precise interpretation of the results.

Our study is subject to several limitations, many of which are inherent to the primary literature. The most significant limitation is the high degree of statistical heterogeneity observed in many of our key analyses, including those for heavy drinking days, liver enzymes, and PTSD symptoms on the PCL scale. This heterogeneity suggests that the included studies varied considerably in terms of design, patient characteristics, intervention protocols, and outcome assessment. While our sensitivity analyses attempted to explore this, the limited number of studies in each analysis constrains the power of meta-regression to definitively identify the sources of this variability. Another limitation is the relatively small number of studies and patients included. Relatively small cumulative sample size for several key outcomes, specifically alcohol craving (*n* = 92) and PTSD symptom severity (*n* = 313) can limit the statistical power to detect a true, modest treatment effect and may increase the risk of publication bias, while the limited number of included trials (< 10 for all outcomes) precluded the use of formal statistical tests for publication bias—such as Egger’s regression or funnel plot asymmetry testing, which lack sufficient power in small samples—we mitigated this risk through a comprehensive search of multiple databases to minimize this risk. Furthermore, the included studies often involved complex patient populations with multiple comorbidities, and the use of NAC as an adjunctive treatment alongside psychotherapy or other medications makes it challenging to isolate the specific effect of NAC. Finally, the duration of treatment in the included trials was often limited, which may be insufficient to observe the full neuroplastic changes and clinical benefits of a glutamate-modulating agent[10].

### Implications, clinical impact, and future directions

From a clinical standpoint, our findings suggest that NAC should not be considered a first-line or standalone treatment for AUD or PTSD based on current evidence. The lack of a consistent, significant effect on primary outcomes of drinking behavior and trauma symptoms means that clinicians cannot expect reliable improvements for the average patient. However, the favorable safety profile (aside from manageable GI effects) and its availability as an over-the-counter supplement mean it could be considered as a tertiary adjunctive option on a case-by-case basis, particularly if other treatments have failed. The signal of efficacy for PTSD in our sensitivity analysis, though not definitive, might encourage clinicians to consider a trial of NAC for patients with non-comorbid PTSD who are seeking supplemental treatment options.

The significant heterogeneity and promising sensitivity analysis findings underscore the need for more rigorous, well-defined trials: future studies should stratify patients using biomarkers (e.g., MRS glutamate imaging, genetic markers) to identify responders and focus on homogeneous groups such as non‐comorbid PTSD, determine optimal NAC dosing and duration through longer trials to evaluate neuroplastic and symptomatic effects, adopt standardized, validated outcome measures to reduce heterogeneity, and address AUD-PTSD comorbidity with trials comparing NAC as an adjunct to trauma-focused versus substance-focused psychotherapies.

## Conclusion

In conclusion, our meta-analysis indicates that N-acetylcysteine, when compared to placebo, does not demonstrate consistent efficacy in reducing the primary symptoms of Alcohol Use Disorder or Post-Traumatic Stress Disorder in broad patient populations. Its therapeutic utility is further limited by a lack of significant impact on associated depressive and anxiety symptoms. However, the treatment is relatively safe, with the primary adverse effect being manageable gastrointestinal distress. A noteworthy signal of potential efficacy for PTSD symptoms emerged in a sensitivity analysis, suggesting that the therapeutic promise of NAC should not be entirely dismissed. Rather than providing a definitive answer, our findings highlight the substantial heterogeneity in the field and underscore the urgent need for more refined, biomarker-guided clinical trials to delineate specific patient subgroups who may yet benefit from this glutamate-modulating agent.

## Electronic Supplementary Material

Below is the link to the electronic supplementary material.

Supplementary Material 1

## Data Availability

All data generated or analysed during this study are included in this published article and its supplementary information file.

## Abbreviations

ALT: Alanine Aminotransferase
AST: Aspartate Aminotransferase
AUD: Alcohol Use Disorder
AUQ: Alcohol Urge Questionnaire
BDI-II: Beck Depression Inventory
CAPS: Clinician-Administered PTSD Scale
CI: Confidence Interval
DASS: Depression Anxiety Stress Scales
DSM: Diagnostic and Statistical Manual of Mental Disorders
GABA: Gamma-Aminobutyric Acid
GGT: Gamma-Glutamyl Transferase
GLT-1: Glutamate Transporter 1
GSH: Glutathione
HADS: Hospital Anxiety and Depression Scale
MD: Mean Difference
NAC: N-acetylcysteine
PACS: Penn Alcohol Craving Scale
PCL: PTSD Checklist
PRISMA: Preferred Reporting Items for Systematic Reviews and Meta-Analyses
PTSD: Post-Traumatic Stress Disorder
RCT: Randomized Controlled Trial
ROB-2: Cochrane Risk of Bias tool 2
RR: Risk Ratio
SD: Standard Deviation
SMD: Standardized Mean Difference
SUD: Substance Use Disorder
TLFB: Timeline Followback
VAS: Visual Analogue Scale
WHO: World Health Organization
