# Supplementary material for: N-acetylcysteine for patients with alcohol use disorder, post-traumatic stress disorder, and their co-occurrence: a systematic review of placebo-controlled randomized trials
Source: BMC Psychiatry. 2026 May 11;26:384. doi: 10.1186/s12888-026-08124-8 (PMC13162469; doi:10.1186/s12888-026-08124-8)
Supplement: Supplementary file 1 — Supplementary Material 1 [file 12888_2026_8124_MOESM1_ESM.docx]

**N-Acetylcysteine for patients with Alcohol Use Disorder and Co-occurring post-traumatic stress disorder**


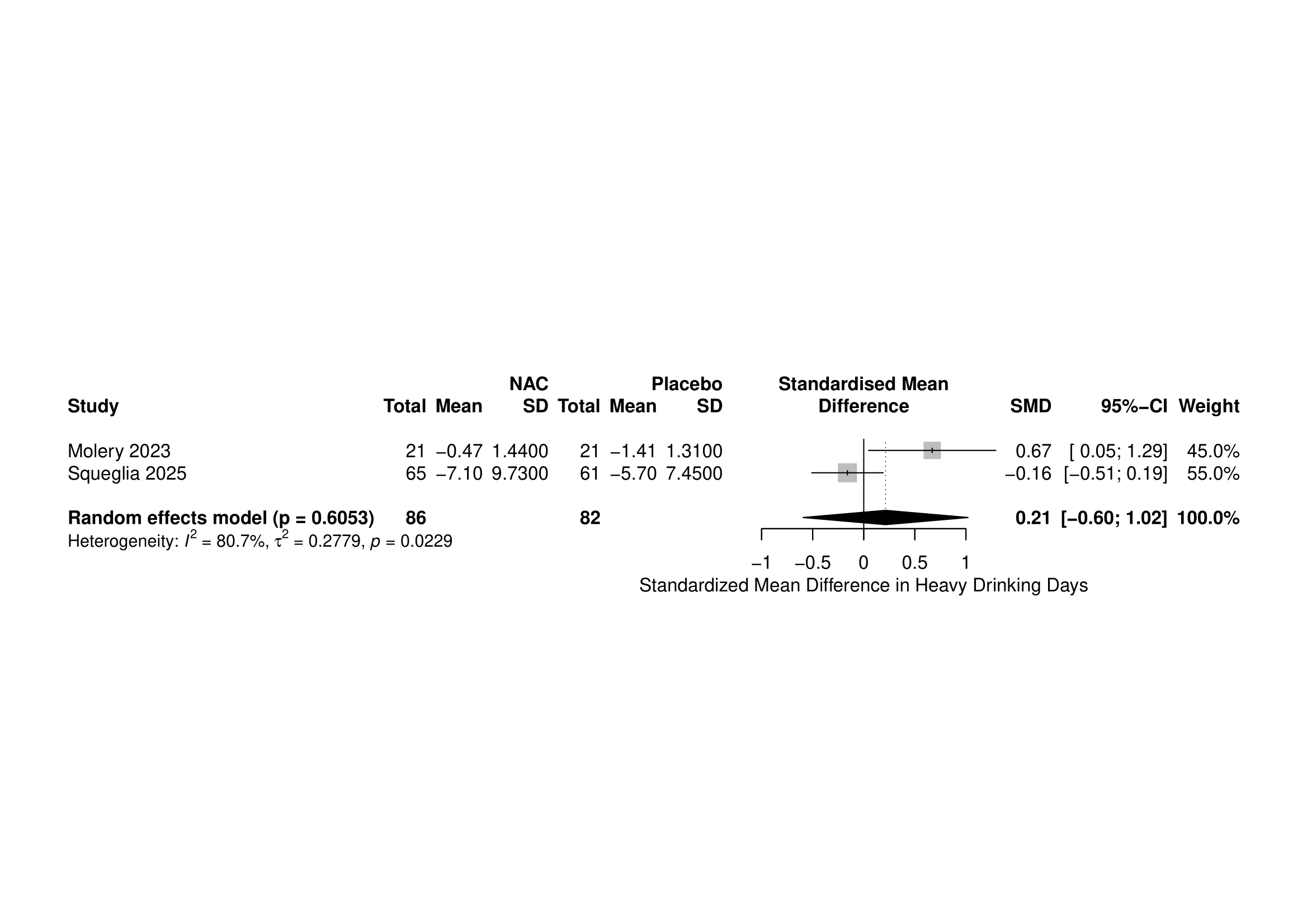


**Figure Supplementary 1.** Forest plot illustrating heavy drinking days


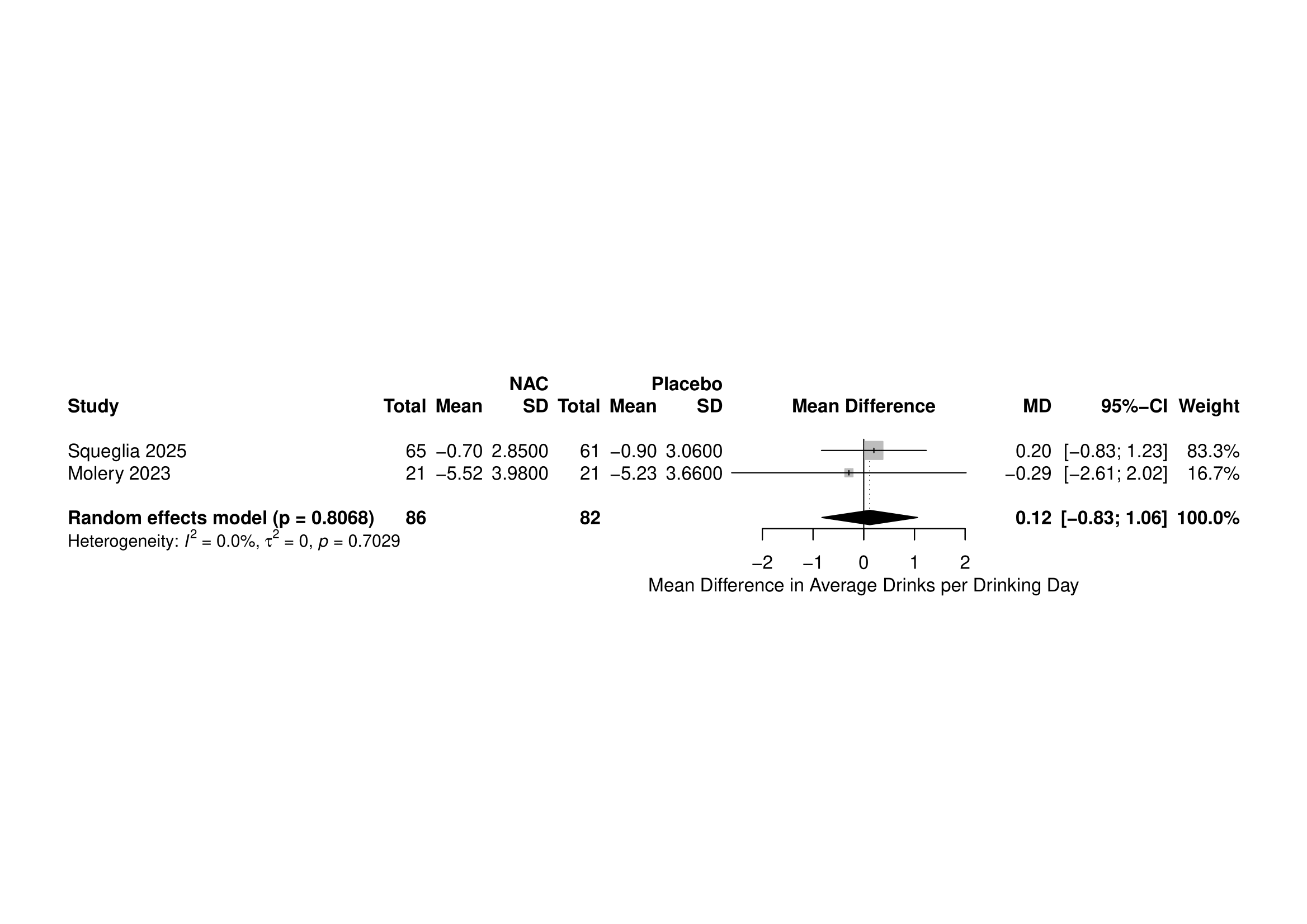


**Figure Supplementary 2.** Forest plot illustrating average drinks per drinking day

**
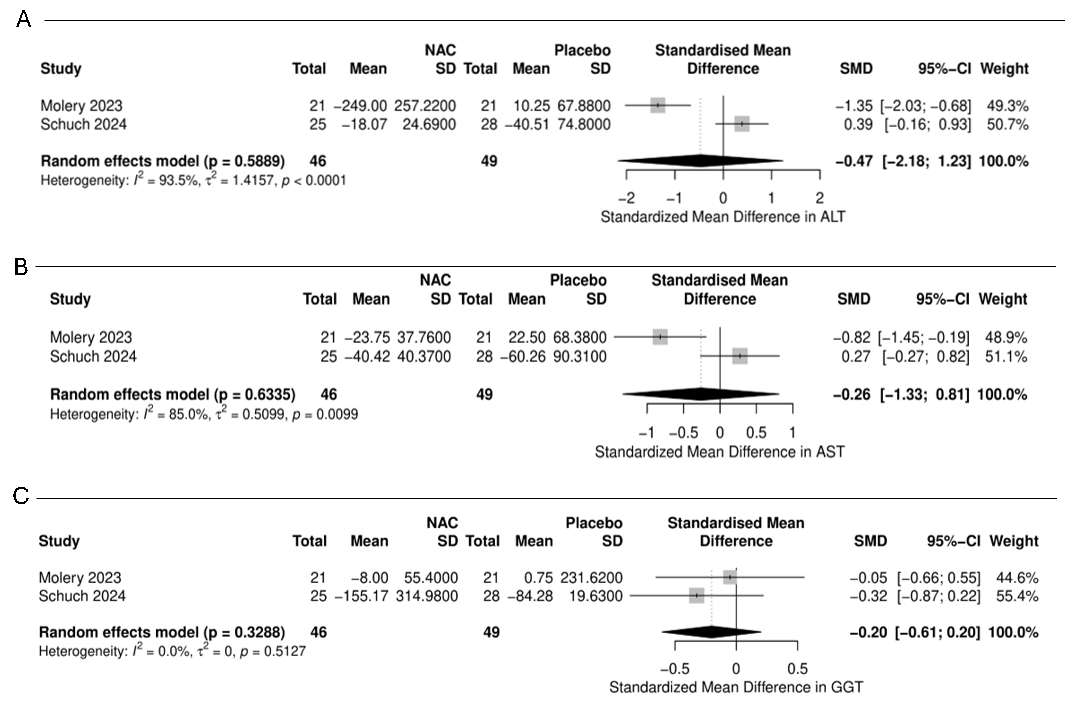
**

**Figure Supplementary 3.** Forest plot illustrating (A) ALT ,(B) AST and (C) GGT


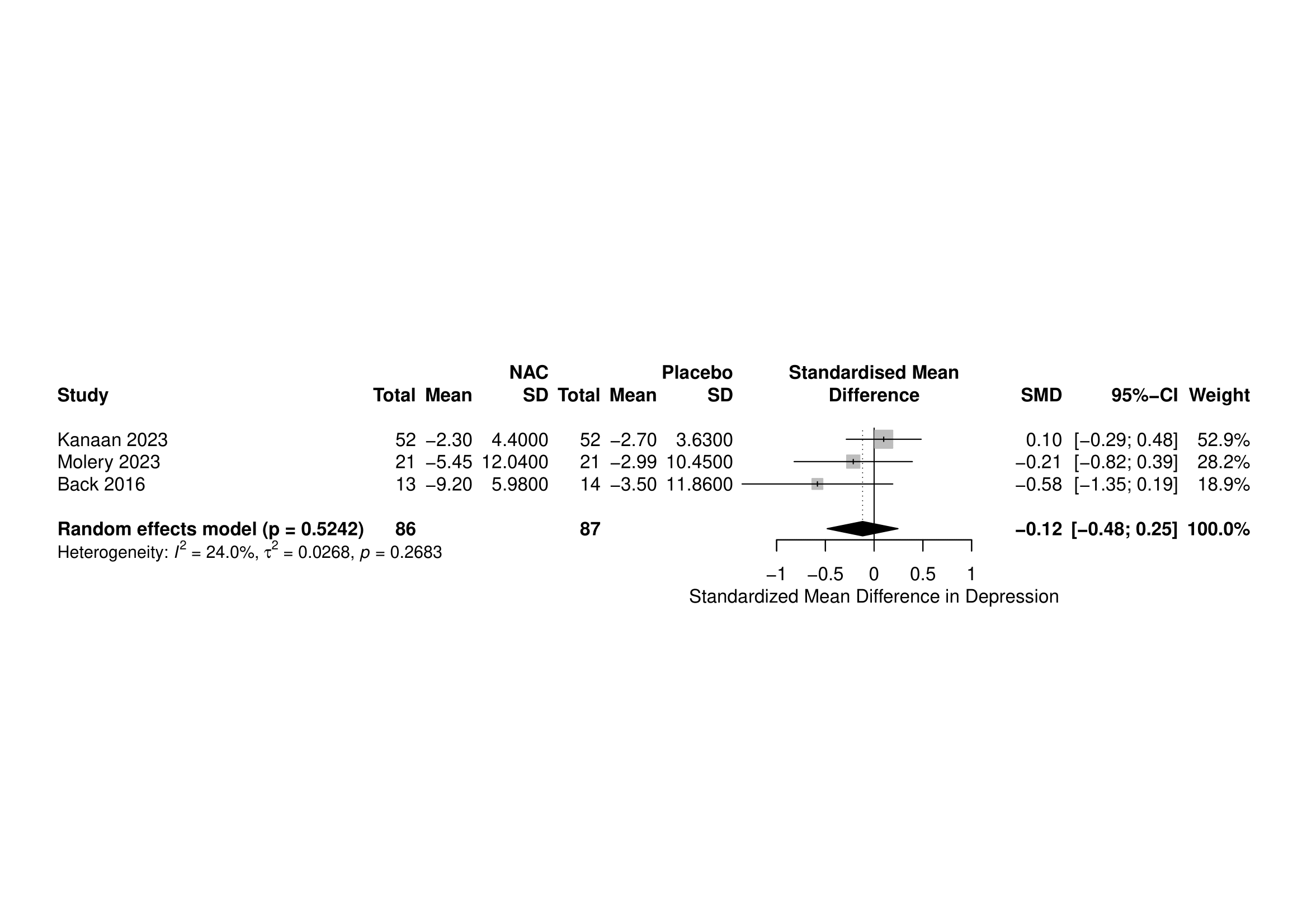


**Figure Supplementary 4.** Forest plot illustrating Depression


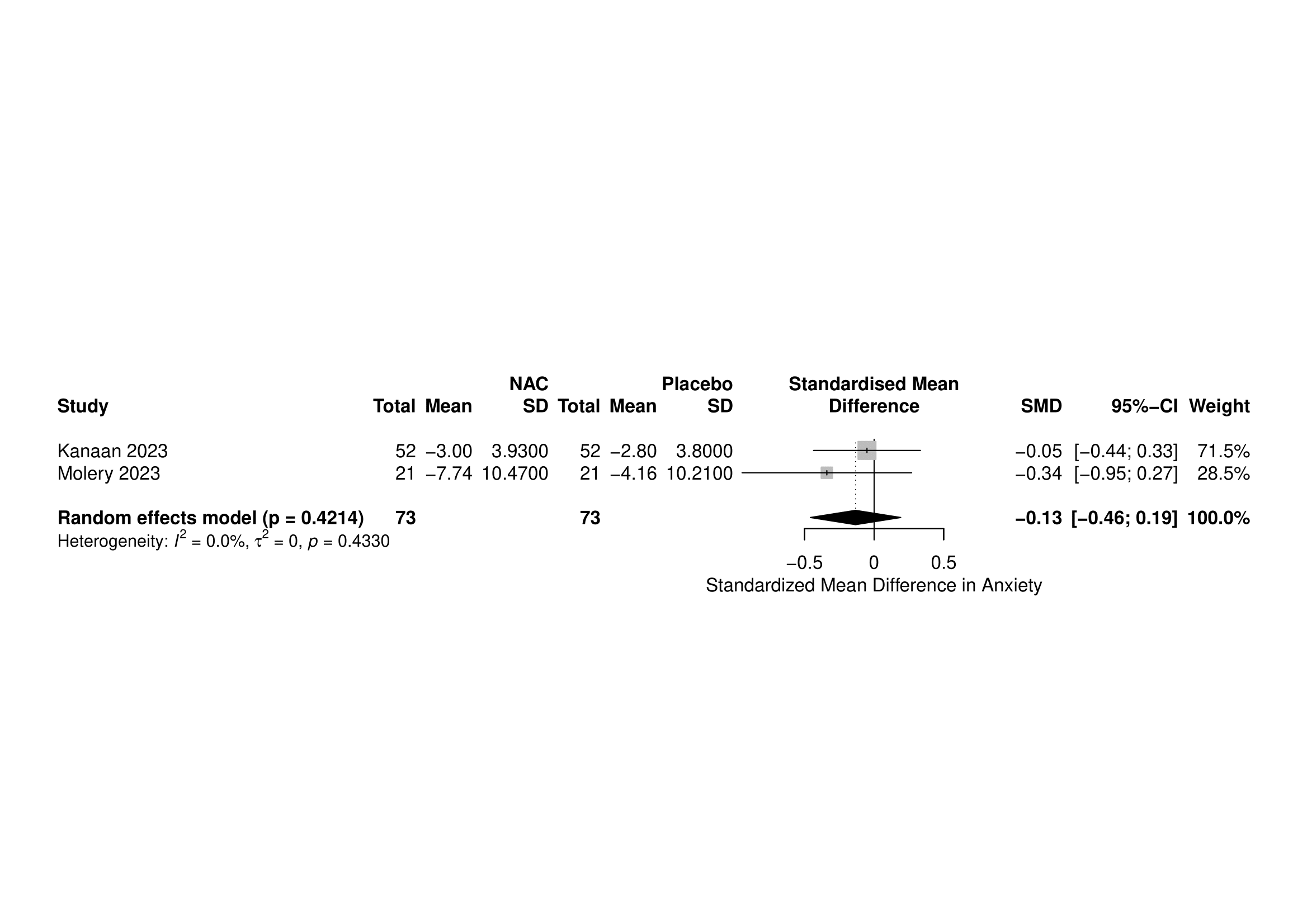


**Figure Supplementary 5.** Forest plot illustrating Anxiety


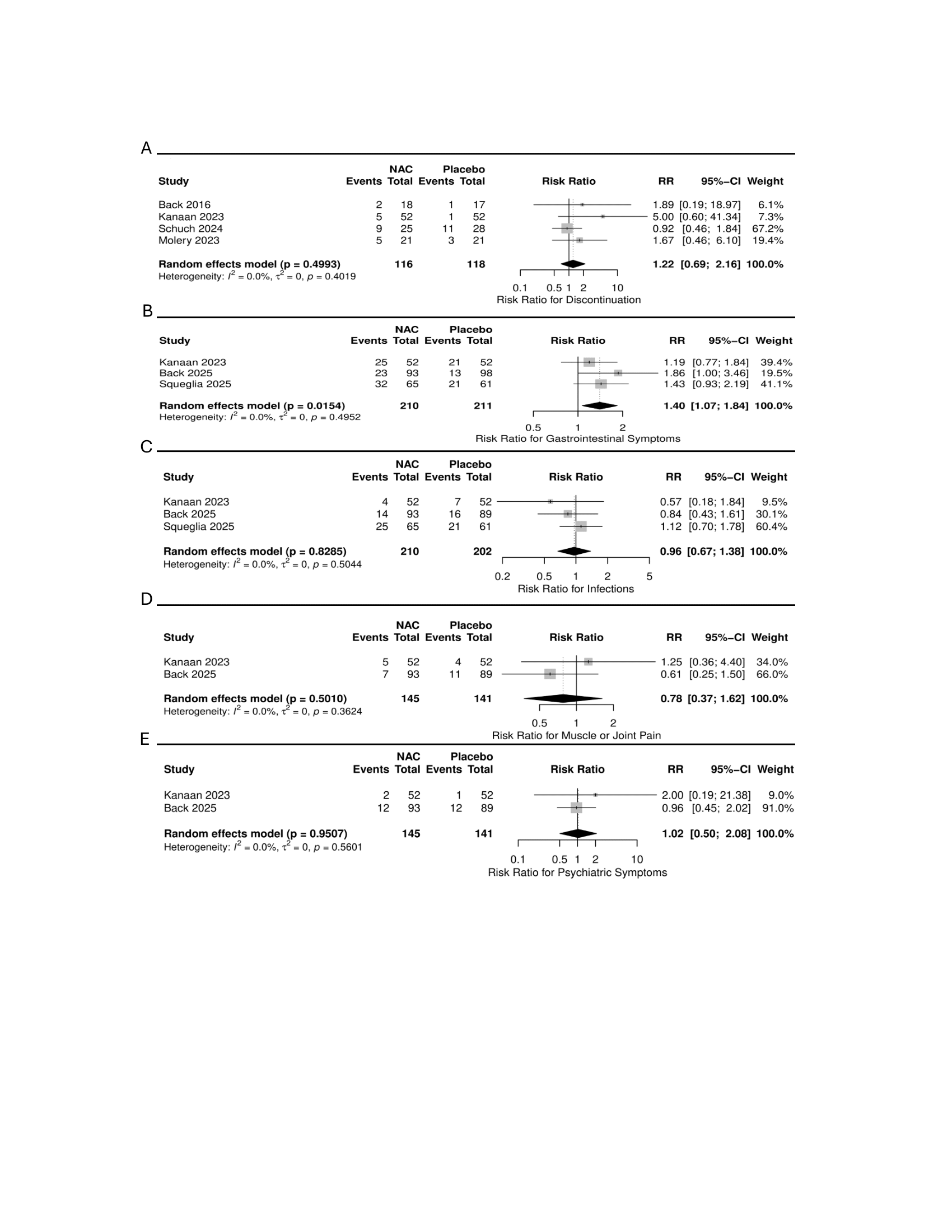


**Figure Supplementary 6.** Forest plot illustrating (A) discontinuation rates ,(B) gastrointestinal symptoms,(C) infections, (D) muscle or joint pain and (E) psychiatric symptoms
